# Study protocol for two pilot randomised controlled trials aimed at increasing physical activity using electrically assisted bicycles to enhance prostate or breast cancer survival

**DOI:** 10.1186/s40814-023-01293-3

**Published:** 2023-04-24

**Authors:** Jessica E. Bourne, Charlie Foster, Chloe Forte, Jonathan Aning, Shelley Potter, Emma C. Hart, Miranda E. G. Armstrong, the CRANK Trial Steering Committee

**Affiliations:** 1 Centre for Exercise, Nutrition and Health Sciences, School of Policy Studies, University of Bristol, 8 Priory Road, Bristol, BS8 1TZ UK; 2 Bristol Urological Institute, Southmead Hospital, North Bristol NHS Trust, Bristol, BS10 5NB UK; 3 Bristol Breast Care Centre, Southmead Hospital, North Bristol NHS Trust, Bristol, BS10 5NB UK; 4 Bristol Medical School, Translational Health Sciences, University of Bristol, 5 Tyndall Avenue, Bristol, BS8 1UD UK; 5 Biomedical Sciences Building, School of Physiology, Pharmacology & Neuroscience, University of Bristol, University Walk, Bristol, BS8 1TD UK

**Keywords:** Prostate cancer, Breast cancer, Electrically assisted cycling, Pilot randomised controlled trial, Physical activity

## Abstract

**Background:**

In 2020, 1.4 and 2.3 million new cases of prostate cancer and breast cancer respectively were diagnosed globally. In the UK, prostate cancer is the most common male cancer, while breast cancer is the most common female cancer. Engaging in physical activity (PA) is a key component of treatment. However, rates of PA are low in these clinical populations. This paper describes the protocol of *CRANK-P* and *CRANK-B*, two pilot randomised controlled trials, involving an e-cycling intervention aimed at increasing PA in individuals with prostate cancer or breast cancer respectively.

**Methods:**

These two trials are single-centre, stratified, parallel-group, two-arm randomised waitlist-controlled pilot trials in which forty individuals with prostate cancer (*CRANK-P*) and forty individuals with breast cancer (*CRANK-B*) will be randomly assigned, in a 1:1 allocation ratio, to an e-cycling intervention or waitlist control. The intervention consists of e-bike training with a certified cycle instructor, followed by the provision of an e-bike for 12 weeks. Following the intervention period, participants in the e-bike condition will be directed to community-based initiatives through which they can access an e-bike. Data will be collected at baseline (T0), immediately post intervention (T1) and at 3-month follow-up (T2). In addition, in the intervention group, data will be collected during the intervention and follow-up periods. Quantitative and qualitative methods will be used. The primary objectives are to determine effective recruitment strategies, establish recruitment and consent rates, adherence and retention in the study, and determine the feasibility and acceptability of the study procedures and intervention. The potential impact of the intervention on clinical, physiological and behavioural outcomes will be assessed to examine intervention promise. Data analyses will be descriptive.

**Discussion:**

The findings from these trials will provide information on trial feasibility and highlight the potential of e-cycling as a strategy to positively impact the health and behaviour of individuals with prostate cancer and breast cancer. If appropriate, this information can be used to design and deliver a fully powered definitive trial.

**Trial registration:**

CRANK-B: [ISRCTN39112034]. CRANK-P [ISRCTN42852156]. Registered [08/04/2022] https://www.isrctn.com.

**Supplementary Information:**

The online version contains supplementary material available at 10.1186/s40814-023-01293-3.

## Background

Prostate cancer (PCa) and breast cancer (BC) are two of the most common cancers globally [1] with 1.4 and 2.3 million new cases diagnosed in 2020 respectively [2]. Improvements in clinical diagnosis and treatment are leading to rising survival rates, and more individuals are living longer with these cancers [3–5]. However, this growing population of cancer survivors experience a range of physical and psychological side effects associated with their cancer and/or treatment and are more likely to suffer from other chronic diseases [6–8]. As such, there is growing interest in developing lifestyle interventions that can help reduce side effects, improve quality of life and prevent further morbidity [9, 10].

Engaging in physical activity (PA) has been identified as having numerous health benefits in cancer survivors [11, 12]. Among PCa survivors, engagement in moderate to vigorous physical activity (MVPA) throughout the cancer journey has been found to lead to improvements in quality of life, physical functioning and reduced cancer-specific fatigue [13–15], while among BC survivors, PA engagement has been associated with improvements in self-esteem and quality of life [16]. Furthermore, higher levels of post-diagnosis PA are associated with reduced risk of all-cause mortality and PCa or BC-specific mortality compared to those who engage in low levels of PA [17–23]. It is therefore recommended that cancer survivors engage in PA during and after cancer treatment [24, 25]. In the UK, NICE now recommend supervised exercise as part of PCa treatment [26]. However, clinical teams are rarely able to deliver targeted PA information or supervised exercise programmes to PCa patients due to time constraints and feeling under qualified to promote or deliver such a programme [27]. Within BC treatment, the promotion of PA is not currently an integrated part of routine care. A UK survey found that around half of oncologists and surgeons do not routinely discuss PA with their BC patients [28]. Furthermore, BC patients report not getting information and/or PA recommendations from healthcare professionals during and after treatment [29].

The lack of PA promotion during treatment is concerning as rates of PA are low among cancer survivors [30–32], and many individuals with PCa and BC fail to meet PA guidelines [33] with self-reported reductions in PA levels following diagnosis and during treatment [34]. Reported barriers to engaging in PA among cancer patients relate to both treatment, including fatigue, pain, a lack of motivation and a decreased confidence to engage in PA and general PA barriers including age-related physical decline and lack of time [34–37]. As such, finding novel ways to increase PA in cancer patients, which increases PA confidence, is a priority.

Electrically assisted bicycles (e-bikes), also known as pedelecs, have been highlighted as a means through which to increase PA in inactive and older adults [38]. E-bikes provide gradated electrical assistance only when the rider is pedalling, through sensors which detect pedalling speed and force. This assistance enables increased speed and reduced physical exertion which is believed to lead to the high level of enjoyment repeatedly reported when e-cycling [39–48] and are likely to be the main motivators that have led to the increased popularity of e-bikes particularly among inactive and older-aged adults [49–51].

Despite the increased assistance, riding an e-bike provides PA of at least a moderate intensity [52–55] and can lead to improvements in cardiorespiratory fitness [55], glucose control [56] and health-related quality of life [57] in inactive or older adults. Among individuals with type 2 diabetes mellitus, e-cycling has been shown to be performed at a moderate intensity with potential for improving glucose control, health-related quality of life and cardiorespiratory fitness [58]. These positive findings suggest that exploring the use of e-bikes as a means of increasing PA in other clinical populations is warranted, including individuals with cancer, a research area yet to be explored. Given the lack of current research, there is insufficient evidence to support full-scale randomised controlled trials (RCT). Rather, pilot RCTs are needed to determine trial feasibility and to provide key information needed for the design of full-scale definitive trials, if warranted.

### Study aims and objectives

The *primary* aim of this study is to test the feasibility of conducting two pilot randomised controlled e-cycling interventions among individuals with PCa (*CRANK-P*) and BC (*CRANK-B*). Two pilot RCTs will be conducted because these different clinical populations have different recruitment and treatment pathways. However, the primary aim of the two trials is the same and will be addressed by answering the following questions:

1. Can individuals with PCa/BC be recruited to an e-cycling trial?
2. Are participants’ willing to be randomised, do they remain in the study and adhere to the intervention and data collection methods, and what are the rates of harmful events?
3. Can the intervention be implemented as intended?
4. Are the intervention and study procedures acceptable to participants, instructors and the clinical team?
5. What are participants’ experiences of e-cycling?

While the pilot RCTs will be insufficiently powered to statistically examine the effectiveness of the intervention on outcomes, they provide an opportunity to investigate the potential promise of the intervention. As such, the *secondary* aim is to examine the association between the intervention and outcome measures to determine intervention promise. To address this aim, the following question will be answered:

6. What is the potential effect of the intervention on a range of individual health and behavioural outcomes?

## Methods

### Study design and setting

**Fig. 1:**
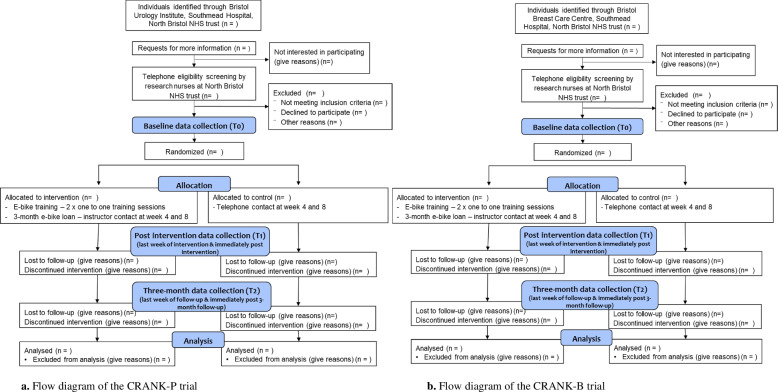
**a** Flow diagram of the CRANK-P trial. **b** Flow diagram of the CRANK-B trial

The proposed trials are single-centre, stratified, parallel, two-arm pilot randomised control trials, whereby individuals with PCa (*CRANK-P*) or BC (*CRANK-B*) will be randomly assigned to either an e-cycling intervention or a standard-care waitlist control. Forty individuals will be recruited for each trial and randomised in a 1:1 allocation ratio to the two study arms. The trials will be conducted in the city of Bristol, England. Recruitment began in June 2022. Measures will be collected predominantly at baseline (time 0 (T0)), immediately following the intervention period (T1) and at 3-month follow-up (T2). In addition, data will be collected in the final week of the e-cycling intervention and follow-up period (PA and travel behaviour) and throughout the intervention and follow-up period (e-cycling time and distance). Figure 1 shows the study flow diagram for the two trials. Reporting of this protocol is according to the SPIRIT checklist (Additional file 1). CRANK-P has been approved by the NHS Health Research Authority Dulwich Research Ethics Committee (REC: 22/LO/0036), and CRANK-B has been approved by the Nottingham Research Ethics Committee (REC: 22/EM/0010). Both trials are sponsored by the University of Bristol. Any amendments to the protocol will be authorised by the sponsor and submitted to the appropriate REC and HRA for approval.

### Recruitment

The two trials will use different recruitment pathways. These are detailed below.

#### CRANK-P

Men who are diagnosed with PCa residing in the Bristol area and who attend the Bristol Urology Institute, North Bristol NHS trust, will be invited to participate. The study will aim to recruit 20 individuals at the start of their cancer journey (i.e. soon after receiving a PCa diagnosis) and 20 individuals who have completed their primary cancer treatment (i.e. after surgery, systemic therapy or radiation therapy) to determine the most appropriate time to recruit individuals during treatment. Patients will be highlighted as potentially eligible at the weekly multidisciplinary team meeting where all patients with a cancer diagnosis are discussed.

For individuals being recruited at diagnosis, initial contact will take place at their diagnosis appointment. At this appointment, patients are informed of their PCa diagnosis by their cancer nurse specialist (CNS). The research will be introduced to the patient at the end of the appointment by saying “We are conducting lifestyle research within the department, are you willing for a research nurse to call you to discuss this further?” This contact will take approximately 1 min. If the patient says yes, they will be provided with an information sheet, and their contact details will be passed on to the urology research nurse team. Men will receive a second opportunity to be informed about the study when they see consultant members of the clinical team (urology/oncology) to discuss their treatment options using the same approach method as above.

For individuals recruited at the end of their primary treatment, initial contact will take place at the patient’s post intensive treatment appointment. At this appointment, patients meet with a CNS or their consultant to discuss their cancer and future treatment. The research will be introduced in the same manner as for those approached at diagnosis, and those who are willing to be contacted will be provided with an information sheet, and their contact details will be passed on to the urology research nurse team.

Individuals who later contact the department interested in participating in the study will have an opportunity to ask questions, an information sheet will be sent by email or in the post, and they will be asked whether they agree to be contacted by a member of the research nurse team after they have had time to read the information sheet.

The urology research nurses will contact individuals who have expressed an interest in participating to confirm eligibility. If eligibility is confirmed, the research nurses will pass on the individual’s contact details to the university research team. All individuals deemed eligible for the study at this point will be invited for baseline testing. Table 1 outlines the enrollment and assessment schedule for the two pilot RCTs.

**Table 1:**
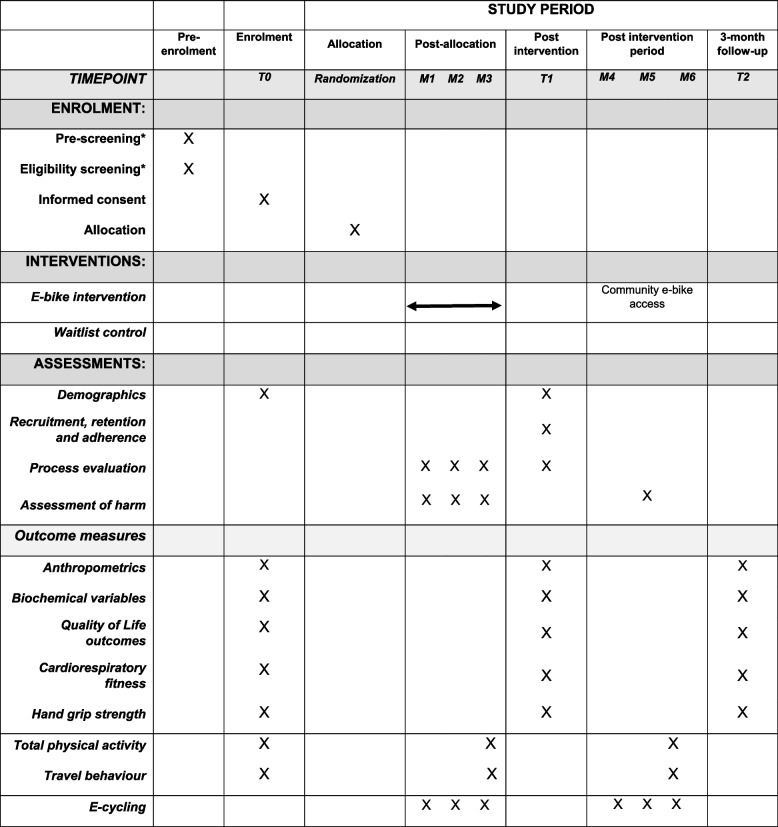
CRANK-P and CRANK-B SPIRIT diagram displaying study recruitment and measures schedule

*Pre-screening and eligibility screening will occur at the Bristol Oncology Centre for CRANK-P and Bristol Breast Care Centre for CRANK-B, T = time, M = month

#### CRANK-B

Individuals who are diagnosed with BC residing in the Bristol area and who attend the Bristol Breast Care Centre, North Bristol NHS trust, will be invited to participate. The study will aim to recruit individuals after their primary cancer treatment (i.e. following completion of surgery, systemic therapy and/or radiation therapy).

At the holistic needs assessment, which occurs after primary cancer treatment, the research will be introduced to the patient by the MacMillan key worker who will ask “We are conducting lifestyle research within the department, are you willing for a research nurse to call you to discuss this further?” If the patient says yes, they will be given a study information sheet, and their contact details will be passed on to the breast care research nurse team.

Individuals who later contact the department interested in participating in the study will have an opportunity to ask questions, an information sheet will be sent by email or in the post, and they will be asked whether they agree to be contacted by a member of the research nurse team after they have had time to read the information sheet.

The BC research nurses will contact individuals who have expressed an interest in participating to confirm eligibility. If eligibility is confirmed, the research nurses will pass on the individuals contact details to the university research team. All individuals deemed eligible for the study at this point will be invited for baseline testing.

The CNS (CRANK-P) and MacMillan key worker (CRANK-B) will keep a record of the number of patients attending the appointments, the number who are deemed potentially eligible and the number of potentially eligible patients who do not wish to be contacted to discuss the research. Individuals who are deemed eligible but who are not interested in participating will be invited to share their decision not to participate if they feel comfortable doing so with the clinical team. The number of individuals not wanting to be contacted and associated reasons, if provided, will be collated and passed on to the research team as a number, so that each refuser remains anonymous, and confidentiality is maintained.

### Data protection

All data collected in these studies will be maintained and stored in accordance with the data protection regulations. All patient identifiable information (i.e. name, contact details, date of birth, home and work postcode) will be stored in a database separate from the database that holds anthropometric measures, results of blood tests, physiological measures and travel and PA data. Personal data stored on NHS, university or Life Cycle computers will be password protected, and only the study investigators or Life Cycle project administrator will have access to the passwords. Personal data on paper files will be stored in a locked filing cabinet within the School of Policy Studies at the University of Bristol or at Life Cycle. All paper files from Life Cycle will be transferred to the University of Bristol. Data will be stored for 15 years.

### Eligibility

The eligibility criteria for the two pilot RCTs are outlined in Table 2.

**Table 2:** Eligibility criteria for the two pilot RCTs

	**Inclusion criteria**	**Exclusion criteria**
CRANK-P	1. Men with D’Amico high-risk localised PCa (*PSA* > 20) or clinical stage ≥ cT2c or Gleason 8/9/10 [59] OR men with any N1 PCa OR men with metastatic PCa (stage M1) 2. Aged 18 years or over 3. Cleared for engaging in PA by the treating consultant	1. Engage in ≥ 150 min of MVPA per week as assessed by the Get Active Questionnaire [60] 2. Uncontrolled hypertension (systolic BP > 160 mmHg and/or diastolic BP > 90 mmHg), for which the individual is not taking medication 3. Comorbidities including myocardial infarction or stroke within the past 6 months or evidence of end-stage renal failure or liver disease, uncontrolled congestive heart failure or angina 4. Use of a mobility aid preventing cycling 5. No previous experience riding a bicycle 6. Any other contra-indications to exercise 7. Are unable to read and communicate in English
CRANK-B	1. Women or men with early BC (i.e. stages 1 or 2) who have completed their primary BC treatment (i.e. surgery, systemic therapy and/or radiation therapy) 2. Aged 18 years or over 3. Cleared for engaging in PA by the treating surgeon	1. Engage in ≥ 150 min of MVPA per week as assessed by the Get Active Questionnaire [60] 2. Individuals with metastatic disease 3. Uncontrolled hypertension (systolic BP > 160 mmHg and/or diastolic BP > 90 mmHg), for which the individual is not taking medication 4. Comorbidities including myocardial infarction or stroke within the past 6 months or evidence of end-stage renal failure or liver disease, uncontrolled congestive heart failure or angina 5. Use of a mobility aid preventing cycling 6. No previous experience riding a bicycle 7. Any other contra-indications to exercise 8. Are unable to read and communicate in English

*BC* breast cancer, *BP* blood pressure, *cT* clinical T category, *N* degree to which cancer has spread to lymph nodes, *M* degree to which cancer has spread to other areas of the body, *MVPA* moderate-to-vigorous physical activity, *NICE* National Institute of Health and Care Excellence, *PA* physical activity, *PCa* prostate cancer, *PSA* prostate-specific antigen

### Sample size

The study will aim to recruit and randomise 40 individuals for each pilot RCT. This sample size is based on recommendations for pilot studies which aim to provide an estimation of a standard deviation for use in the sample size calculation to inform a larger randomised controlled trial [61, 62], as well as the availability of e-bikes. There are no explicit targets regarding the number of individuals to be recruited or screened as we are investigating the feasibility of recruitment from the clinical setting. Based on recruitment rates in a similar population (individuals recovering from colorectal cancer) and recruitment occurring in a clinical setting, we anticipate a recruitment rate of approximately 20% [63]. Recruitment and screening will close when 20 participants have been randomised to each of the two study arms. Based on a previous pilot randomised controlled trial of e-cycling among individuals with type 2 diabetes mellitus, a retention rate of approximately 80% is anticipated [58].

### Consent

Once participants have been identified as eligible to participate in the studies, they will be booked in for a baseline data collection visit (T0). At this visit, a member of the research team will outline the study procedures, as per the information sheet. Participants will be advised that the study is voluntary, and that they have the right to withdraw at any time, without the need for explanation. After this, individuals who wish to participate will be asked to read, complete and sign a consent form, which will be countersigned by the member of the research team obtaining consent.

### Allocation, randomisation and blinding

Randomisation will occur after consent is obtained and baseline data (T0) has been collected. For each pilot RCT, 40 individuals will be randomly assigned to either the e-cycling intervention or waitlist control in a 1:1 allocation ratio. For CRANK-P, individuals will be stratified based on cancer treatment received prior to randomisation. For CRANK-B, individuals will be stratified based on stage of cancer treatment prior to randomisation. Permuted blocks of random size will be used. A random allocation sequence will be generated by an independent statistician using R v4.1.2, package blockrand (v1.5). The randomisation sequence will be accessible through a password-protected Excel file. The researcher will access the Excel file and allocate the participant to either condition in the order issued in the sequence. Researchers will be aware of the group allocation. Participants will be informed of the group allocation via telephone by a member of the research team. Blinding of intervention allocation will not be possible for any participant involved in the trial or the researchers.

### Intervention and control groups

#### Intervention development and content

The development and content of the intervention is being prepared for publication (Bourne et al., The development of a theory and evidence-based intervention to increase physical activity in individuals with prostate cancer and breast cancer, the CRANK trials. Manuscript in preparation). Briefly, the intervention was developed in accordance with the MRC guidance for the development of complex interventions [64] and the Behaviour Change Wheel [65]. A programme theory, identifying the theoretical underpinnings of the intervention was developed using theory, evidence and patient and public involvement (PPI). After identifying the hypothesised mechanisms of change, intervention content was guided by the selection of specific behaviour change techniques (BCTs) using the 93-item behaviour change technique taxonomy (BCTTv1) [66]. A total of twenty-three BCTs were identified for inclusion.

The mode of intervention delivery was based on previous feasibility work examining the feasibility of an e-cycling intervention in adults with type 2 diabetes mellitus delivered by the same community organisation [67]. Once developed, the proposed intervention was presented to members of the PPI group consisting of cycling instructors, cancer patients and experts in PA behaviour change. Feedback was elicited through open discussion and in written form. Intervention content, including instructor manuals and participant workbooks, were adapted accordingly. The final intervention content and delivery modes are presented in Additional file 2 and described below.

#### Instructor training

The intervention will be delivered by a Bristol-based charity who specialise in bicycle training, Life Cycle. Instructors are fully qualified National Standard cycle instructors, and so training, provided prior to delivery, will focus on the behavioural aspects of the intervention content. Training will consist of two sessions. Training session 1 (3 h) will provide information on importance of PA for individuals with cancer and the general physical and mental health benefits of engaging in PA. Motivational interviewing techniques will be taught to help instructors engage with participants, and information on the specific content of the intervention will be provided. The use of motivational interviewing has been found to support physical activity behaviour change in cancer patients [68, 69]. Training session 2 (4 h) will provide an opportunity to review the intervention content. Role-playing activities will be conducted and feedback provided from the researchers. Attendance at the training is mandatory for delivering the intervention. Instructors will be observed during initial intervention delivery sessions, and feedback will be provided by the research team. A bi-monthly peer support group will be run enabling instructors to share their experiences of delivering the intervention.

#### E-bike training

Following baseline measures (T0), participants allocated to the intervention will complete e-bike training at Life Cycle. Training will consist of two one-to-one sessions. Training session 1 will be mandatory and will follow the bikeability guidelines for levels 1 and 2. Individuals’ previous cycling experience will be considered when conducting the cycling-specific training. Training session 2 will occur within 2 weeks of session 1. The instructor and participant will discuss the need and/or desire for session 2. Session 2 will provide participants with an opportunity to practice e-cycling skills with the instructor. Training sessions 1 and 2 will last approximately 2 h each. All participants must have completed bikeability level 2 prior to taking the e-bike home.

Throughout these sessions, the instructors will deliver evidence-based behaviour change techniques through motivational interviewing informed conversations. Participants will be provided with a workbook to record notes from discussions with instructors and their e-cycling goals throughout the intervention.

Participants will be encouraged to monitor their e-cycling using either a GPS watch (Fitbit Charge 5) or a paper logbook for the duration of the intervention. If using the GPS watch, participants will have the option to pair the watch with a mobile application to monitor their e-cycling.

Participants will be invited to join a private social media group, one group for each pilot RCT, to share their experiences and ride ideas with other individuals participating in the intervention. At the end of the e-bike training, the participant will be free to take the e-bike home and use as they wish. E-bikes can be ridden home or bike transportation provided by Life Cycle. Upon taking the e-bike home, participants will be provided with maps of cycle routes in the area, instructions of a call out maintenance service in case of breakdown, helmet, pannier, bike lock and lights.

#### E-bike loan

Participants will be loaned the e-bike for 12 weeks for their personal use. Participants will be instructed to use the e-bike as they desire, with no specific daily or weekly cycling frequency or distance targets imposed by the researchers. Throughout the loan period, participants will be invited to attend both study-specific e-cycling group rides run bi-monthly and pre-existing group rides run monthly by Life Cycle. At weeks 4 and 8 of the loan, participants will participate in one-to-one refresher sessions with their instructor (sessions 3 and 4). These sessions will take place at a location of the participant’s choice and last approximately 2 h each. The content of the session will depend on the participant’s needs but will include a review of participants e-cycling progress so far, action planning and goal setting for future riding and practicing riding on established or new routes. At the end of week 12, participants will return the e-bike to Life Cycle, or an instructor will collect the e-bike.

#### 3-month follow-up

Following completion of the intervention, individuals in the e-bike condition will be provided with information, in the form of a booklet, on how to access community-based e-cycling initiatives. This will include how to access the cycle2work scheme, information on community e-bike trial sessions, community e-bike loan availability and e-bike discounts. In addition, a pool of five e-bikes for each trial will be available for participants to loan for 12 weeks from the University of Bristol. These e-bikes will be allocated on a first come, first served basis. Individuals’ decision to seek out e-cycling opportunities at this time will be at their own discretion. Exploring the current available pathways through which individuals can access an e-bike in the community is important for exploring future programme sustainability. Throughout this time, individuals in the e-bike condition will be asked to record any e-cycling activity using the GPS watch and paper logbook.

#### Control group

Individuals assigned to the waitlist control after baseline data collection (T0) will receive two phone calls from the researcher at approximately weeks 4 and 8 to maintain engagement in the study. During these phone calls, the researcher will ask participants about how their cancer treatment is going and will direct participants to cancer support groups in line with standard care procedures. Between post-intervention data collection (T1) and 3-month follow-up data collection (T2), individuals will not be contacted. After the 3-month follow-up, individuals in the control condition will be offered training session 1 and loaned an e-bike for 3 months. Sessions 2, 3 and 4 will not be conducted. Participants will be asked to report any contact they have with other individuals in the study to ensure no contamination between conditions has occurred.

### Data collection

#### Demographics

Information regarding age, gender, ethnicity, home and work postcodes will be collected at baseline. The English Index of Multiple Deprivation (IMD; Department for Communities and Local Government, 2015) [70] based on full home postcode will be determined as a measure of community socioeconomic status (SES). Cancer stage at diagnosis and treatment pathway information will be obtained from the clinical team.

#### Feasibility and acceptability

To assess feasibility of recruitment, the number of individuals that attend the clinics and the number that are deemed eligible will be recorded. Recruitment rates, consent rates and willingness to be randomised will be recorded. Retention rates will be determined based on the number of individuals that complete the intervention, post testing, and follow-up measures. Adherence rates to study procedures will be recorded.

The acceptability of the recruitment methods and individuals’ receptiveness to the study will be explored through semi-structured one-to-one interviews with the clinical care team. The acceptability of the study procedures, including data collection methods, and the intervention will be explored through semi-structured one-to-one interviews of all study participants and instructors. These interviews will be conducted by a member of the research team. Interview questions for instructors will focus on factors that impact intervention delivery, including intervention content, facilities, time and burden. Interview questions for participants will focus on thoughts and feelings regarding participation in the intervention and data collection processes. The research team will track the costs and resources required for running the trial. Life Cycle will track the staff costing of intervention delivery and from the maintenance service. This information will be shared with the University of Bristol research team.

#### Intervention fidelity

The degree to which the intervention was delivered and received as intended (implementation) will be evaluated following criteria proposed by Lambert and colleagues [71]. Specifically, all instructors delivering the intervention will undertake training which will include information on the benefits of PA for individuals with cancer and training on how to engage in discussions around personal health. A separate training session focused on the theoretical bases of the intervention, the principles of motivational interviewing and the specific intervention content will also be delivered. Training will include role plays with supervised feedback which will be documented. Following the training, instructor’s intervention delivery will be observed by a member of the research team and observation checklists completed to record instructors’ delivery of the intervention content, receipt of the intervention by participants and enactment of the behavioural skills. Throughout the intervention, instructors will be asked to complete checklists to ascertain what skills training and discussions occurred during each session.

Intervention intensity, recorded by instructors, will be determined through recording of the number of intervention sessions attended by participants as well as the volume of email and telephone contact between instructors and participants. At the end of the study, information on attendance to intervention sessions and tasks completed within each session will be passed from Life Cycle to the research team. Participant’s ID, and not names, will be recorded on intervention documentation.

#### Process evaluation

To explore the mechanisms of impact, semi-structured interviews will be conducted with participants in the intervention group to identify barriers and enablers to engaging in e-cycling. The interviews will be guided by the Theoretical Domains Framework (TDF) to descriptively link the identified barriers and facilitators to pathways of behaviour change. The TDF is an integrative framework that synthesises 33 psychological theories and 128 constructs to understand the broad influences on behaviour. The TDF is an elaboration of the COM-B model, an evidence-based model identifying three key sources that interact to influence behaviour, namely capability, opportunity and motivation [65]. These interviews will help to identify how the intervention impacts behaviour and to determine contextual factors that influence the intervention.

#### Assessment of harm

Participants will be asked to report adverse events resulting from e-cycling (e.g. musculoskeletal problems, falls or road traffic incidents) by calling the study phone line. All adverse events will be immediately documented in the study master file and if required reported to the study sponsor and appropriate NHS trust as per standard procedures. Each month, the number and types of adverse events that have taken place will be shared with the Trial Steering Committee to determine whether it is appropriate for the trial to continue. Adverse events that mean the participant is unable to continue with the intervention will also be documented under retention rates. Qualitative interviews will be used to explore any unintended consequences that arise from participation in the study.

#### Outcome measures

Unless stated otherwise, outcome measures will be assessed at baseline (T0), immediately post-intervention (T1) and at 3-month follow-up (T2) and will be collected in both trials.

##### Anthropometrics

Participants will be asked to take off shoes and remove heavy clothing. Body weight will be assessed to the nearest 0.1 kg using digital scales (TANITA Corp, Tokyo, Japan), and height will be assessed to the nearest 0.1 cm (SECA, 700 SECA Hamburg, Germany). These measures will be used to calculate BMI (kg/m^2^). Waist circumference will be measured using a non-stretch tape measure to the nearest 0.1 cm, based on World Health Organization guidelines [72]. Waist circumference measurements will be taken twice, and the average of those measures calculated.

##### Biochemical variables

Blood samples will be obtained by venepuncture of the antecubital fossa from individuals in a fasted state (≥ 8-h overnight fast) to measure glucose, insulin, insulin-like growth factor (IGF) I and II, IGF-binding protein 2 and IGF-binding protein 3. In CRANK-P, prostate-specific antigen (PSA) will also be measured. A total of 8 mL of blood will be taken at this time.

All blood samples will be transported, using a sealed container, immediately to the biochemistry laboratory at the Bristol Royal Infirmary where they will be centrifuged and the serum aliquoted prior to freezing at − 80 °C. Samples will be batch analysed at the end of the study. Glucose, insulin and lipids will be analysed using a Roche Cobas C701 analyser (Roche Diagnostics, Rotkreuz, Switzerland). IGF-I, IGF-II and IGFBP-3 will be analysed using radioimmunoassay, and IGFBP-2 will be analysed using enzyme-linked immunosorbent assay (ELISA). PSA will be analysed using a Roche Cobas C602 analyser. Insulin, glucose, lipids and PSA will be analysed at the USTAR laboratory. IGF factor samples will be transported to the Metabolic Endocrinology Group at the Learning and Research Building, Southmead, North Bristol, where they will be analysed. Basal insulin and glucose values will be used to calculate insulin resistance and beta-cell function using the homeostasis model assessment calculator (University of Oxford, Diabetes, Trial Unit).

##### Generic health-related quality of life (HRQoL)

The EuroQol-5 dimension-5 level survey (EQ-5D-5L) will be used to measure generic quality of life. The measure classifies health into five dimensions (mobility, self-care, usual activities, pain/discomfort, anxiety/depression) with five response categories including no problems, slight problems, moderate problems, severe problems and unable to/extreme problems [73]. Responses are coded as single-digit numbers which express the severity level selected in each dimension in a descriptive manner to generate a health state profile. From this, a health state index score will be calculated from individual health profiles using the England-specific value set [74]. The 5Q-5D-5L also includes a vertical visual analogue scale for respondents to self-assess their overall health based on ‘how good or bad your health is today’ with responses ranging from 0 to 100, where 100 represents ‘the best health you can imagine’. This measure has been widely used to assess generic health-related quality of life in cancer populations in response to exercise interventions [75–77] and is the most widely used measure of HRQoL internationally [78].

##### Cancer-specific health-related quality of life

The European Organisation for Research and Treatment of Cancer Core Quality-of-Life Questionnaire (EORTC QLQ-C30) will be collected to assess cancer-specific HRQoL. In addition, in CRANK-P, the 25-item PCa-specific (EORTC PR-25) questionnaire module will be collected, while in CRANK-B, the 45-item BC-specific (EORTC BR-45) questionnaire module will be collected. The EORTC QLQ-C30 is a 30-item questionnaire developed to assess general cancer-related aspects of quality of life covering the most common cancer symptoms (pain, fatigue, nausea, vomiting) and functional outcomes including physical, role, social, emotional and cognitive functioning. Items are scored on a 4-point Likert scale ranging from 1 (not at all) to 4 (very much), and a raw score is calculated for each multi-item scale. Raw scores are linearly transformed to a 0 to 100 scale for global health, functionality and symptoms [79]. The EORTC QLQ-C30 is an internationally validated measure and is the most widely used questionnaire for the assessment of HRQoL in cancer clinical trials [80–82]. Higher functional scores represent better functioning, a high score for global health status represents a higher quality of life and higher symptom scores indicate more severe symptoms.

The EORTC PR-25 is a 25-item PCa-specific questionnaire module designed to supplement the EORTC QLQ C-30 [83] and will be used in the current study to gather more PCa-specific HRQoL information. The PR-25 is composed of one single-item measure and five multi-item subscales to provide four symptom scores (urinary symptoms [eight items], bowel symptoms [four items], hormonal treatment-related symptoms [six items], incontinence aid [one item]) and two functional scores (sexual activity [two items] and sexual functioning [four items]). PR-25 sexual function items are completed only by those respondents who have been sexually active over the preceding 4 weeks. Items are scored on a 4-point Likert scale ranging from 1 (not at all) to 4 (very much), and a raw score is calculated for each multi-item scale. Raw scores are linearly transformed to a 0 to 100 scale. Higher functional scores represent better functioning, and higher symptom scores indicate more severe symptoms.

The EORTC BR-45 is a 45-item BC-specific questionnaire module designed to supplement the EORTC QLQ C-30 and will be used in the current study to gather more BC-specific HRQoL information. The BR-45 is composed of five function scores (body image [four items], future perspective [one item], sexual functioning [two items], sexual enjoyment [one item], breast satisfaction [two items]) and seven symptom scores (systemic therapy side-effects [seven items], upset by hair loss [one item], arm symptoms [three items], breast symptoms [four items], endocrine therapy symptoms [ten items], skin/mucosa symptoms [six items], endocrine sexual symptoms [four items]). Items are scored on a 4-point Likert scale ranging from 1 (not at all) to 4 (very much), and a raw score is calculated for each multi-item scale. Raw scores are linearly transformed for functional scales and symptom scales ranging from 0 to 100. As with PR-25, higher scores reflect more symptoms or higher levels of functioning. The BC-specific module of the EORTC is considered the standard instrument for measuring QoL in patients with both early and metastatic BC [84, 85].

##### Urinary health (CRANK-P)

The International Consultation on Incontinence Questionnaire Male Lower Urinary Tract Symptoms Module (ICIQ-MLUTS) will be used to evaluate male lower urinary tract symptoms [86, 87]. This measure assesses the prevalence and bother of 13 urinary symptoms as they were experienced on average over the past 4 weeks. Prevalence is scored on a scale from 0 (never) to 4 (all of the time), while bother of each of the 13 symptoms is scored on a scale of 0 (not at all) to 10 (a great deal). The questionnaire contains two predefined domains (adding up the scores of individual items): a void domain (maximum score 20 for prevalence and 50 for bother) and an incontinence domain (maximum score 24 for prevalence and 60 for bother). This measure has been found to be a valid and reliable instrument for evaluating lower urinary tract symptoms in men [87].

##### Erectile dysfunction (CRANK-P)

The International Index of Erectile Function (IIEF) will be used to measure erectile dysfunction. This 15-item measure is designed to diagnose the presence and severity of erectile dysfunction. Each item is scored from 1 to 5 or 0 to 5, and summary scores are created for the 4 domains of male sexual function: erectile function (maximum score 30), orgasmic function (maximum score 10), sexual desire (maximum score 10) and intercourse satisfaction (maximum score 15) and two questions relating to overall satisfaction (maximum score 10) [88]. The IIEF has been reported to be a reliable and valid measure of erectile dysfunction [89] and is the most widely used measure within clinical settings to measure erectile dysfunction [90].

##### Fatigue

The Functional Assessment of Chronic Illness Therapy-Fatigue (FACIT-F) measure will be used to assess cancer-related fatigue. FACIT-F is a 13-item scale that asks respondents to rate statements regarding their fatigue experiences and its impact on their daily lives. Sample items include the following: ‘I feel fatigued’, ‘I feel weak all over’ and ‘I feel listless’. Items are measured on a 4-point Likert scale from 0 (not at all) to 4 (very much). Items are summed to provide an overall score ranging between 0 (severe fatigue) and 52 (no fatigue) [91]. This item was designed specifically for use with cancer patients [92].

##### Self-efficacy for coping with cancer

The Cancer Behaviour Inventory-Brief version (CBI-B) is a 12-item measure of self-efficacy expectations about coping with cancer [93]. Participants report their level of confidence for the items on a Likert scale from 1 (not at all confident) to 9 (totally confident). Items are summed to provide a general estimate of coping efficacy, with higher scores indicating a higher degree of self-efficacy in coping with cancer. This measure has been found to be a reliable and valid measure of self-efficacy [93, 94] and is one of the most widely used measures of self-efficacy for coping with cancer [95].

##### Cardiopulmonary exercise testing (CPET)

At baseline, all participants will be screened for cardiac contraindications for exercise testing using a resting ECG to rule out unstable cardiac disease (e.g. acute myocardial infarction and unstable arrhythmia). The resting ECG will be reviewed immediately by a cardiac consultant or registrar who will provide the final say as to whether an individual is cleared to complete the CPET. Resting ECGs will be conducted at baseline, and CPET clearance will stand for the entirety of the trial. For individuals who are cleared to complete the CPET, the peak volume of oxygen (O_2_) consumed and used (VO_2peak_) will be assessed using an incremental ramped maximal exercise test on an upright Ergoselect bicycle ergometer (Ergoselect 100, Love Medical, Manchester, UK). Two researchers will be present during the testing. The test will begin with a 5-min baseline period, while the participant is sat upright on the cycle ergometer and resting respiratory values assessed (via breath-by-breath gas exchange monitoring and spirometry; Ergostik CPET system, Love Medical, UK). The participant will begin with a warm-up of unloaded cycling for 3 min. Following the warm-up, the resistance will increase continuously, V̇O_2_ peak will be assessed using an incremental ramped protocol of 10 to 25 W/min until volitional fatigue. Participants will be asked to maintain a constant cadence of 60–80 revolutions per minute (RPM). Participants will be provided with positive encouragement through the assessment and encouraged to ‘keep pushing’ towards the end of the assessment. The test will be terminated upon volitional exhaustion or when the cadence falls below 60 rpm. The increase in work rate was chosen to bring the participant to the limit of tolerance within 8 to 12 min. VO_2_peak will be defined as the highest 15-breath moving average for VO_2_ (in absolute [l/min] and relative [ml/kg/min] terms. Heart rate (12-lead ECG), oxygen saturation (ear pulse oximeter), blood pressure (automated brachial arm cuff) and rating of perceived exertion (Borg scale 6–20) will be assessed throughout CPET.

##### Hand-grip strength

Participant’s hand-grip strength will be measured on each hand using a hydraulic hand dynamometer (Jamar Hand Dynamometer, USA) which can measure isometric grip force up to 90 kg. This device has been found to have good reliability and reproducibility [96]. The device will be calibrated at the start of each measurement day. Each participant will be asked to sit upright on a chair with their forearm on the armrest and then grasp the handle of the device in their right hand. Participants are required to maintain a 90° angle in their elbow adjacent to their side so that their thumb faces upwards while squeezing the handle as strongly as possible for approximately 3 s. The same protocol will be applied to the left hand. The measure will be repeated three times on each hand. Standardised encouragement will be provided during the assessment. The average values of the two hands will be used.

##### Total PA behaviour

PA behaviour will be assessed at baseline and in the last week of the 12-week intervention and 3-month follow-up period using the Axivity AX3 wrist-worn triaxial accelerometer (Axivity, Newcastle, UK). Participants will wear the waterproof device for seven consecutive days, for 24 h a day, on their nondominant wrist. Raw acceleration files (.cwa), with a sampling frequency of 100 Hz, will be downloaded using the OmGui Software (Open Movement, Newcastle, UK) and processed in R software using the software package GGIR [97]. Specifically, the package will convert raw accelerations (*x*-, *y*- and *z*-axes) to magnitudes of dynamic acceleration (expressed as Euclidean Norm Minus One, ENMO) averaged over 1-s epochs in milli-gravitational units (m*g*). Time accumulated in MVPA per week will be calculated and expressed in milli-gravitational units (m*g*). Thresholds of approximately 100 mg and 400 mg will be used to represent moderate and vigorous intensity activities respectively [98]. Participants will be required to have at least three valid days of data according to standard GGIR software package processing to be included in the analysis. Non-wear time will be defined as consecutive stationary episodes lasting for at least 60 min. The outputs will be processed to provide time spent in MVPA per week. These monitors have been validated to assess PA energy expenditure [99].

##### Travel behaviour (changes in transportation modes and trip purpose)

Spatial location data will be collected using a personal GPS receiver (Qstarz International Co. Ltd., Taiwan) to describe participants travel behaviour at baseline (i.e. before obtaining the e-bike), in the final week of the intervention and in the final week of the 3-month follow-up. Participants will be asked to wear the GPS during waking hours and recharge it at night. The device can be worn around the waist or in a pocket as desired. GPS time and positional data will be used to identify journey start and finish times and estimate velocities. In addition, participants will also be asked to complete a 7-day travel diary over the same period as GPS monitoring. The travel diary is adapted from Neves and Brand [100] and will ask participants to record every journey made over the 7-day period. Participants will be asked to classify the journey under one of five categories: commuting for work, commuting for education, travel for business, shopping or personal business and social visits or leisure activities. For each journey, participants will be asked to report the travel mode, start and end time and start and end location. The information from the travel diary will be used to (a) capture contextual data related to trips (e.g. trip purpose), (b) validate the GPS and (c) provide an alternative source of data in case the GPS fails, is not turned on, or the battery dies. This information will allow us to determine the total distance travelled by active transportation (i.e. walking or cycling) compared with motorised transportation and to compare different modes of active transportation.

##### E-cycling behaviour and intensity

E-cycling behaviour will be measured during the intervention and follow-up period in the intervention group using a Fitbit Charge 5 watch. The Fitbit Charge 5 is an integrated GPS and heart rate monitoring watch which can store up to 7 days of activity tracking. Participants will have the option to pair the device with a smart phone using Bluetooth. If Bluetooth pairing is used, then activity data will be automatically uploaded to the Fitbit website. If the participant chooses not to pair the device, then the data will be downloaded each week by the researcher. The watch will need charging during the loan period; all charging cables and instructions will be provided. Outcomes derived from the watch will include the frequency and duration of e-cycling journeys and intensity of activity associated with e-cycling based on heart rate data. Wrist-worn consumer devices, including the Fitbit, have been reported to have acceptable accuracy for the measurement of heart rate in research settings and are more ecologically valid than research devices [101, 102]. Average journey speed will also be derived. The purpose of journeys will be derived through land use maps and using home and work postcodes. Participants will be provided with a paper logbook should they not wish to use the watch. The logbook will ask participants to record each e-cycling trip, the duration, the start and end location and the purpose. An inbuilt odometer on each bike will also collect data on the total number of kilometres ridden during the trial in case the watch or logbook is not used. Participants will use the watch or complete the logbook for the intervention period and for the 3-month follow-up period. After this time, the watches will be returned to the researcher, and data from the Fitbit device and/or online website will be downloaded to a University of Bristol computer and securely stored. After all data is downloaded, it will be deleted from the Fitbit website and the device itself. Participants will not be required to enter their name, address or email when setting up the Fitbit device; all devices will be connected to a University of Bristol email address, and the participant’s ID will be used in place of their name. However, due to the nature of GPS tracking, participant’s start and end points of journeys will be identifiable from the GPS tracks. This will be made clear to the participants in the information sheet and in the consent form.

### Data analysis

#### Quantitative analysis

The primary outcomes of this pilot trial include recruitment and consent rates, retention and adherence to study procedures and data provision. Analysis of these data will be descriptive, expressed as frequencies and percentages. Any adverse events will be described appropriately. Characteristics of the sample will be summarised using descriptive statistics (means and standard deviations, medians and interquartile ranges or frequencies and percentages as appropriate). Descriptive comparisons of these data will be made between the intervention and the waitlist control. Evidence of promise of the intervention (i.e. whether the intervention can lead to changes in outcomes measures) will be examined using comparison of change scores between conditions for all outcome measures (except e-cycling during the intervention). See Table 3 for a description of the outcome measures and proposed analysis plan for each outcome. Effect estimates will be presented with 95% confidence intervals reported; *p*-values will not be considered as the study is not powered to detect effectiveness. All analysis will be undertaken in Stata v17.

**Table 3:** List of CRANK research questions, associated outcomes, data collection tools, time point measurements and analysis plan

Research question	Outcome	Data collection method/tool	Time point of measurement				Analysis plan
			Baseline (T0)	During intervention	Post (T1)	3-month FU (T2)	
1. Can individuals with prostate/breast cancer be recruited into an e-cycling trial?	• No. of individuals in clinic; no. of meeting eligibility criteria; no. of express interest in participating • Reasons for not wanting to participate in the study • No. of individuals that consent to be part of the study	Study records	X				Frequencies and percentages
2. Are participants’ willing to be randomised, do they remain in the study and adhere to the intervention and data collection methods and what are the rates of harmful events?	• No. of participants retained in study following randomisation • No. of individuals that complete follow-up testing • No. of participants that attend each of the intervention sessions and data collection sessions • No. of harmful events	Study records		X			Frequencies and percentages
3. Can the intervention be implemented as intended?	• No. of training sessions attended by participants and additional contact with instructors • Extent to which intervention content is completed as planned • No. of and extent of adaptations	Intervention checklists		X			Frequencies and percentages
4. Are the intervention and study procedures acceptable to participants, instructors and clinical team?	• Acceptability of recruitment strategy to clinical team • Acceptability of intervention to participants • Acceptability of study procedures to participants • Acceptability of intervention delivery to instructors	Semi-structured interviews			X		Thematic analysis based on research question
5. What are participants experiences of e-cycling?	• Experiences of e-cycling • Participant’s barriers and facilitators to e-cycling	Semi-structured interviews			X		Thematic analysis based on research question
6. What is the potential effect of the intervention on a range of individual health and behavioural outcomes?	• Weight, BMI and waist circumference	Tanita digital scales, SECA 700, non-stretch take	X		X	X	Comparison of change scores between conditions Reporting of effect estimates with 95% CI
	• Metabolic markers (fasting glucose, insulin, insulin resistance, lipid profile, IGF-I, IGF-II, IGFBP-2, IGFBP-3, PSA^a^)	8-mL blood sample	X		X		
	• General health-related quality of life	EuroQol-5 dimension-5 level	X		X	X	
	• Cancer-specific quality of life	EORTC QLQ-C30 EORTC PR-25^a^ EORTC BR-45^b^	X		X	X	
	• Urinary health^a^	ICIQ-MLUTS	X		X	X	
	• Erectile dysfunction^a^	International Index of Erectile Function	X		X	X	
	• Fatigue	FACIT-F	X		X	X	
	• Self-efficacy for coping with cancer	Cancer Behaviour Inventory — Brief	X		X	X	
	• Cardiorespiratory fitness	Maximum oxygen uptake using cycle ergometer	X		X	X	
	• Hand grip strength	Hydraulic hand dynamometer	X		X	X	
	• Total physical activity (time spent in moderate-to-vigorous physical activity)	Axivity AX3	X		X	X	Comparison of change scores between conditions
	• Travel behaviour • No. of trips • Transport mode (walking, cycling, e-cycling, car, bus, train) • Trip purpose	QStarz GPS and travel diary	X		X	X	Mean and SD
	• E-cycling behaviour and intensity: no. of journeys, duration, distance travelled, purpose of use	Bike odometer, Fitbit Charge 5 GPS watch, logbook		X			Mean and SD

^a^CRANK-P only, ^b^CRANK-B only

#### Qualitative analysis

Recordings of interviews will be transcribed verbatim. Data will be analysed using the framework method [103] and guided by Gale and colleagues seven-stage analysis process [104]. This process will involve both deductive and inductive coding based on the research questions. Throughout the process, each transcript will be analysed independently by two researchers. Once complete, the two researchers will compare and discuss coding and categorisation. Any disagreements will be discussed and resolved through consensus.

## Discussion

This paper describes the protocol of *CRANK-P* and *CRANK-B*, two pilot randomised waitlist-controlled trials designed to evaluate the feasibility and acceptability of an e-cycling intervention for individuals with PCa or BC. PA is an important component of cancer treatment, however PA behaviour tends to decrease following a cancer diagnosis [34]. Finding novel and effective ways of engaging individuals with cancer in PA is essential to promote positive health outcomes. E-cycling provides at least a moderate intensity PA with the potential to positively impact health [55]. The potential impact of e-cycling among individuals being treated for PCa or BC has yet to be explored. Research is needed to examine the *acceptability* of e-cycling in these populations, to determine the *feasibility* of conducting a randomised controlled trial and to determine if e-cycling demonstrates *potential to positively impact both health and behavioural outcomes*. The current e-cycling intervention has been developed using previous research, theory and engaging with PCa or BC patients, instructors and experts in behaviour change. The proposed study has some limitations. Firstly, there may be potential selection bias when approaching potential participants, whereby the clinical team may make judgements about the suitability of the intervention for a specific patient. To overcome this potential bias, all recruiting staff members will be trained on the inclusion and exclusion criteria. Secondly, the lack of blinding may create challenges with study retention particularly in the control group. This is common to many exercise studies, and this has been addressed in the current study by offering all control participants e-bike training and a 12-week loan at the end of the trial. Thirdly, the single-centre pilot trial limits the ability to generalise to other cities across the UK or rural areas in which the feasibility and associated outcomes could be different.

Despite these limitations, the data collected in this trial will provide insight into the acceptability of an e-cycling intervention for individuals with cancer and to ascertain whether such a trial is feasible. This information is necessary to inform the development of future e-cycling interventions and identify appropriate outcome measures for examination in a definitive trial if deemed appropriate.

## Supplementary Information

**Additional file 1.** SPIRIT 2013 Checklist: Recommended items to address in a clinical trial protocol and related documents*.

**Additional file 2.** Content of the CRANK intervention.

## Data Availability

Not applicable.

## Abbreviations

BC: Breast cancer
BCT: Behaviour change technique
BMI: Body mass index
METs: Metabolic equivalents
MVPA: Moderate-to-vigorous physical activity
PA: Physical activity
PCa: Prostate cancer
RCT: Randomised controlled trial
