# Supplementary material for: Study protocol for two pilot randomised controlled trials aimed at increasing physical activity using electrically assisted bicycles to enhance prostate or breast cancer survival
Source: Pilot Feasibility Stud. 2023 Apr 24;9:68. doi: 10.1186/s40814-023-01293-3 (PMC10124052; doi:10.1186/s40814-023-01293-3)
Supplement: Supplementary file 2 — Additional file 2. Content of the CRANK intervention. [file 40814_2023_1293_MOESM2_ESM.docx]

| **Additional File 3.** Content of the CRANK intervention | | |
| --- | --- | --- |
| **Session** | **Intervention content** | **Associated behaviour change technique** |
| **E-bike training phase** | | |
| Session 1  Mandatory face to face | One-to-one physical training on e-bike with Life Cycle UK instructor including using gears, how to ride in traffic etc. Training will follow Bikeability Level 1 and if appropriate Level 2. Instructor to demonstrate the different skills followed by the participant practicing them. | 4.1 Instruction on how to perform behaviour  6.1 Demonstration of behaviour  8.1 Behavioural practice/rehearsal |
|  | Instructor to provide participant with feedback on their e-cycling (e.g., on technique, form etc.) | 2.2 Feedback on the behaviour |
|  | Introduce participant to their workbook and the general information in there that they can refer to. Show participant how to use the logbook and encourage them to record their activity. Also introduce the participant to the Garmin Watch as an additional/alternative form of tracking behaviour. If desired help the participant set up the watch and connect to the Garmin Connect App on their phone. | 2.3 Self-monitoring of behaviour |
|  | Instructor to provide participant with information on potential health and emotional benefits associated with physical activity and specifically e-cycling, particularly in regard to cancer and reducing feelings of fatigue. The potential environmental consequences of e-cycling will also be discussed. | 5.1 Information about health consequences  5.4 Information about emotional consequences  5.3 Information about social and environmental consequences |
|  | Encourage participants to identify potential barriers to e-cycling and come up with ways to overcome these barriers and record these in their workbook for next time | 1.2 Problem solving |
| Session 2  Mandatory face to face | One-to-one physical training on e-bike with Life Cycle instructor following Bikeability Levels 2 and 3 if appropriate. Instructor to demonstrate the different skills followed by the participant practicing them. Ensure skills build on difficultly from previous session if appropriate for the participants skill level | 4.1 Instruction on how to perform behaviour  6.1 Demonstration of behaviour  8.1 Behavioural practice/rehearsal  8.7 Graded tasks |
|  | Instructor to provide participant with feedback on their e-cycling (e.g., on technique, form etc.) | 2.2 Feedback on behaviour |
|  | Prompt participant to practice the skills they have learnt in quiet locations/roads and then increasing length/frequency or duration of rides as they get more confident | 8.7 Graded tasks |
|  | Participant encouraged to record their e-cycling in the logbook or using the Garmin Watch. | 2.3 Self-monitoring of behaviour |
|  | Review and discuss participants identified barriers to e-cycling. Chat about strategies identified to overcome them. | 1.2 Problem solving |
|  | Work with the participant to identify an e-cycling goal (this should be as specific as possible including e.g., frequency, duration, purpose, where and when the behaviour will be completed etc.). Record goal in workbook | - 1. Goal setting (behaviour)   1.4 Action planning |
|  | Advise participant to plan their e-bike journeys and collect all the gear they need in one location ahead of time to increase the likelihood of conducting the activity and/or forgetting something | 7.1 Prompts/cues  11.3 Conserving mental resources |
|  | Participant provided with helmet, panniers, and lights for the duration of the e-bike loan period and cycling maps. Participant provided with details of bike breakdown service which can be utilized throughout the intervention period. | 12.5 Adding objects to the environment  3.2 Social support (practical) |
|  | Participant connected to other participants via social media (WhatsApp Group) and provided with information on Life Cycle UK social rides and cancer group specific rides | 3.2 Social support (practical) |
|  | Participants also advised to connect with friends and family and to inform them of their goals to build support | 3.3 Social support (emotional) |
|  | If participants are feeling anxious about e-cycling in general or with traffic encourage them to try riding in quiet locations to practice before trying out busier locations. Help them to identify where and when they may do this. | 11.2 Reduce negative emotions |
| **E-bike loan phase** | | |
| Session 3  Face-to-face  Location of participants choice | Participant and instructor ride together. Participant to decide on where they would like to practice riding – could involve trying a new route or trying a busy road. Instructor to provide instruction if required and demonstrate skills. Participant to practice these during the session. | 4.1 Instruction on how to perform behaviour  6.1 Demonstration of behaviour  8.1 Behavioural practice/rehearsal |
|  | Instructor to provide feedback to the participant on their riding | 2.2 Feedback on behaviour |
|  | Instructor and participant to review past 4-weeks e-cycling behaviour using logbook/Garmin watch. Instructor to provide positive encouragement about capabilities and to encourage participant to focus on past success. | 2.2 Feedback on behaviour  15.1 Verbal persuasion about capabilities  15.3 Focus on past success |
|  | Review behaviour and how these fits with goals set during training. Revise goals if appropriate. Record new goals in workbook. | 1.5 Review behaviour goals |
|  | Encourage participant to switch one car journey/public transport journey for an e-bike ride and to plan how and when they will do this ride. | 8.4 Habit reversal  1.4 Action planning |
|  | Review barriers to e-cycling that have arisen and how these were overcome/could be overcome in the future | 1.2 Problem solving |
|  | Participant encouraged to plan where and when they want to ride in the future. | 1.4 Action planning |
|  | If participants are feeling anxious about e-cycling in general or with traffic encourage them to try riding in quiet locations to practice before trying out busier locations. Help them to identify where and when they may do this. | 11.2 Reduce negative emotions |
| Session 4  Face-to- face Location of participants choice | Participant and instructor ride together. Participant to decide on where they would like to practice riding – could involve trying a new route or trying a busy road. Instructor to provide instruction if required and demonstrate skills. Participant to practice these during the session. | 4.1 Instruction on how to perform behaviour  6.1 Demonstration of behaviour  8.1 Behavioural practice/rehearsal |
|  | Review of e-cycling behaviour over the past month and instructor to provide feedback | 2.2 Feedback on behaviour |
|  | Instructor and participant to review past 4-weeks e-cycling behaviour using log book/Garmin watch. Instructor to provide positive encouragement about capabilities and to encourage participant to focus on past success. | 2.2 Feedback on behaviour  15.1 Verbal persuasion about capabilities  15.3 Focus on past success |
|  | Review behaviour and how these fits with goals set during training. Revise goals if appropriate. Record new goals in workbook. | 1.5 Review behaviour goals |
|  | Review of barriers that have arisen and discussion on how these were overcome/plan ways to overcome these | 1.2 Problem solving |
|  | Discuss how and where participants plan to ride in the final 4 weeks | 1.4 Action planning |
|  | Recap on the potential health and emotional benefits participant may experience through e-cycling | 5.1 Information about health consequences  5.4 Information about emotional consequences |
